# Change in 1-year mortality after hip fracture surgery over the last decade in a European population

**DOI:** 10.1007/s00402-022-04719-4

**Published:** 2022-12-02

**Authors:** Francisco A. Miralles-Muñoz, Adolfo Perez-Aznar, Santiago Gonzalez-Parreño, Emilio Sebastia-Forcada, Gerard Mahiques-Segura, Alejandro Lizaur-Utrilla, M. Flores Vizcaya-Moreno

**Affiliations:** 1 Department Orthopaedic Surgery, Elda University Hospital, Ctra Elda-Sax S/N, 03600 Elda, Alicante Spain; 2 Department Traumatology and Orthopaedia, Miguel Hernandez University, Avda Universidad S/N, 03202 San Juan de Alicante, Alicante Spain; 3 Clinical Research Group, Faculty of Health Sciences, University of Alicante, Ctra San Vicente del Raspeig, S/N, 03690 San Vicente del Raspeig, Alicante Spain

**Keywords:** Hip fracture, Elderly, Mortality, Risk prediction, Epidemiology

## Abstract

**Objective:**

There are scarce data on the mortality after hip fracture surgery for patients treated in the most recent years. The objective of this study was to analyze whether the overall initiatives introduced over the last decade for elderly patients with hip fractures had a positive impact on the 1-year mortality.

**Methods:**

Patients treated during 2010–2012 were compared with patients treated during 2018–2020 for all-cause 1-year mortality. Variables influencing mortality were collected based on the literature, including demographic, comorbidity, cognitive status, and preinjury physical function. Crude mortalities were compared between periods, as well as with the expected mortality in the general population adjusted for age, gender, and year of surgery using the standardized mortality ratio (SMR). A multivariate model was used to identify mortality risk factors.

**Results:**

591 patients older than 65 years were treated during 2010–2012 and 642 patients during 2018–2020. The mean age increased significantly between periods (78.9 vs. 82.6 years, respectively, *p = *0.001) in both genders, together with an increase in comorbidity (*p = *0.014). The in-hospital mortality risk had no significant difference between periods (2.5 vs. 2.0%, *p = *0.339), but the 30-day mortality risk (8.3 vs. 5.5%, *p = *0.031) and 1-year mortality risk (16.1 vs. 11.9%, *p = *0.023) declined significantly. However, 1-year mortality in 2020 had an excess of 1.33 in SMR. Age older than 80 years, male gender, and Charlson comorbidity index > 2 were significant predictors of 1-year mortality.

**Conclusion:**

The important evolution achieved in the last decade for the management of patients with hip fracture surgery has led to a significant decline in 1-year mortality, but the 1-year mortality remains significantly higher compared to the general population of similar age and gender.

## Introduction

Hip fractures remain associated with high mortality in the elderly population, and they represent an important public health concern with significant financial cost implications [1]. During the last decades, advances in anesthesia and surgical procedures emerged with the aim of reducing surgical mortality risks. However, as older patients and with more comorbidities are currently undergoing surgery, the postoperative complications and especially mortality risk remain a serious concern [2].

Despite these advances, the 1-year mortality after hip fracture surgery is reported as high as 29% [3], which represented an excess 3–4 times higher than expected in the general population [4]. Few studies on secular trends and changes in excess mortality after hip fractures over time are available and the results are conflicting [3, 5, 6]. While some studies reported no significant mortality risk changes from1960 to 2000 in United Kingdom [3] and from 2000 to 2014 in Denmark [5], other more recent study found a significant decline from 2000 and 2016 in Italy [6]. Most of these large studies were based on national registries that included all patients with hip fracture older than 18 or 50 years [5, 7]. On the other hand, most studies available reported trends for mortality after hip fracture including historical cohorts of the 1990s or data from patients treated before 2005, and they do not consider that care has evolved in the last 10 years [3].

With the implementation in the last decade of specific hip fracture pathways [8], such as early surgery, preoperative optimization of patients at higher risk and multi-disciplinary team between surgeons and geriatricians for perioperative and postoperative cares of these elderly patients [9], one would argue that there would probably be an improvement in morbidity and mortality risks. However, data on recent trends in mortality after hip fracture surgery are scarce. Only some studies have focused on patients treated in the last years. Trevisan et al. [6] reported no significant change in 1-year mortality from 2000 to 2016. Likely, Gjertsen et al. [10] reported no significant change from 2005 to 2014, and Kjaervik et al. [11] found small survival differences from 2014 to 2018. Conversely, Nordstrom et al. [12] reported that short-term mortality increased during 1998–2017. Thus, there is scarce evidence on the postoperative mortality or whether the difference in mortality between hip fracture patients and the general population has changed over the last decade.

The objective of this study was to investigate if there was any change in mortality over the last decade. The hypothesis was that improvement in mortality should have occurred.

## Materials and methods

A prospective registry-based observational cohort study was performed after approval by the institutional ethics committee. Informed consent was not required as it was performed on available anonymized data. The level-I trauma department database at our regional public hospital prospectively collected patient data from admission to 1-year postoperatively.

To analyze the mortality trend during the last decade, two periods of three consecutive years each were compared: 2010–2012 and 2018–2020. Patients admitted for hip fracture in these periods were identified from the departmental database. The inclusion criteria were diagnosis of proximal femoral fracture, age 65 years or older, and surgical treatment. For patients with subsequent hip fracture during the study period, only the first fracture was included for study. The exclusion criteria were patients treated with a conservative approach (usually decision to palliative care), pathological fracture (metastasis), and multiple trauma. Patients who were tested positive for an infection by the SARS-CoV-2 virus were also excluded.

During the last 5 years (2016–2020), a co-management of elderly patients between orthopedic surgeons and geriatricians was used from admission to 30-day after discharge. Our strategy for hip fracture was to perform surgery as early as possible, within 48 h from the trauma. All surgeries were performed under spinal anesthesia. Trochanteric fractures were treated with sliding hip screw or intramedullary trochanteric nail when a more stable construct was required, subtrochanteric fractures with locked long intramedullary nail, and cervical fractures with hemiarthroplasty or total hip arthroplasty (usually for active patients under 70 years of age). Postoperative rehabilitation was carried out with the assistance of a physiotherapist, and usually began within 24 h after surgery with mobilization out of bed to a chair. Progressive full weight-bearing with walker was authorized at 48 postoperative day if they tolerated.

### Follow-up and evaluations

All patients were evaluated at admission and up to 1 year postoperatively. The primary outcome was the postoperative all-cause mortality over time (in-hospital, at 30 days, and at 1 year). Our center is linked to all primary healthcare centers, remaining hospitals of our country, and the national mortality register. Data from these different sources could be combined using the unique personal identification number of each citizen, and it is possible to construct the complete medical history for each patient and to identify admission in other outside hospitals.

Variables influencing mortality were collected based on the literature [2, 9] including gender, body mass index (BMI), fracture type (cervical or trochanteric), and time to surgery after admission. Age was also categorized into two groups: 65–79 years and 80 or over. Comorbidity at admission was assessed by the American Society of Anaesthesiology (ASA) (I–II, low risk; III–IV, high risk) [13], and the Charlson comorbidity index (≤ 2 low risk, > 2 high risk) [14]. Cognitive status at admission was measured by the Hodkinson's abbreviated mental test 0–10 score, where six or less suggested dementia [15]. Preinjury physical function was assessed using the Katz Index [16] for activities of daily living (ADL), where full independence was defined as the ability to do all six ADL without assistance, partial dependence as the ability to do four or five activities without assistance, and total dependence as the ability to do three activities or fewer without assistance.

### Statistical analysis

Statistical analyses were conducted with SPSS v. 21 software (IBM, Armonk, USA). A *p* value of 0.05 or less was considered as significant. Kolmogorov–Smirnov tested normal distribution. All variables had a non-Gaussian distribution, and only nonparametric tests were used. Categorical data were analyzed by Fischer’s exact test or Mantel–Haenszel test, and continuous variables by Mann–Whitney test. Independent factors influencing mortality were analyzed by Cox proportional hazards regression models. In the models, significant covariates in univariate analyses were entered, as well as those suggested by the literature as potential predictors. Risks were presented as hazard ratio (HR) with 95% confidence interval (CI).

Crude 1-year mortality was expressed as proportion (%), and also compared with the expected mortality based on the general population adjusted for age, gender, and year of surgery. This last outcome was expressed as the standardized mortality ratio (SMR, ratio of observed to expected deaths) with 95% CI. An SMR > 1 indicated an observed mortality higher than expected. Mortality tables from the National Institute of Statistics were used to determine the expected mortality [17].

## Results

### Patient characteristics

There were 1233 patients who met the criteria. The 2010–2012 cohort was composed of 591 patients, and 2018–2020 of 642 patients. Baseline characteristics of both cohorts are shown in Table 1. There was a significant higher proportion of females throughout the period studied (*p = *0.023), with an increment of males in 2018–2020. In this last period, the mean age increased significantly in both genders (*p = *0.001) with a significant increase in the proportion of patients older than 80 years (*p = *0.033). Likewise, there was a significant increase in comorbidities according to the Charlson index (*p = *0.042), and ASA showed a significant higher proportion of class III–IV patients (*p = *0.023) in 2018–2020 compared with 2010–2012. On the other hand, the proportion of patients operated within 48 h after admission was significantly higher in 2018–2020 (*p = *0.029). Overall, the commonest comorbidities were arterial hypertension (65%), heart failure (27%), diabetes (29%), chronic pulmonary disease (14%), cerebrovascular disease (9%), cancer (8%), hypothyroidism (4%), chronic renal disease (4%), and Parkinson (3%).

**Table 1:** Baseline characteristics of patients

Variables	2010–12 (*n = *591)	2018–20 (*n = *642)	*p* value
Gender, F/M (*n*)	445/146	450/192	0.023*
Females	445 (75%)	450 (70%)	
Males	146 (25%)	192 (30%)	
Age			
Overall (yr)	78.9 (7.8)	82.6 (8.6)	0.001*
65–79 years (*n*)	420 (71%)	424 (66%)	0.033*
≥ 80 years (*n*)	171 (29%)	218 (34%)	
BMI (kg/m^2^)	30.1 (5.4)	29.9 (4.8)	0.491
Charlson			
Overall (points)	2.7 (2.8)	3.1 (2.9)	0.014*
≤ 2 (*n*)	254 (43%)	244 (38%)	0.042*
> 2 (*n*)	337 (57%)	398 (62%)	
ASA class (*n*)			0.023*
I–II	402 (68%)	401 (62%)	
III–IV	189 (32%)	241 (38%)	
Mental index (points)	7.6 (3.4)	7.2 (3.8)	0.052
Katz index (points)	4.4 (1.5)	4.2 (2.2)	0.064
Fracture (*n*)			0.074
Trochanteric	349 (59%)	402 (63%)	
Subtrochanteric	35 (6%)	41 (6%)	
Cervical	208 (35%)	199 (31%)	
Time to surgery			0.029*
Overall (days)			
< 48 h (*n*)	395 (67%)	462 (72%)	
> 48 h (*n*)	196 (33%)	180 (28%)	

Continuous data as mean (SD)

*Significant *p* value

### Mortality analysis

The commonest causes of death throughout the study period were pneumonia (31%) and cardiac failure (22%), without substantial differences between both periods. Comparing 2010–2012 and 2018–2020 cohorts with univariate analyses (Table 2), the in-hospital mortality risks were 2.5% and 2.0%, respectively (*p = *0.339), but 30-day (8.3% and 5.5%, *p = *0.031) and 1-year (16.1% and 11.9%, *p = *0.023) mortalities declined significantly. The cumulative 30-day mortality risk declined significantly in females from 8.8% in 2010–2012 to 5.5% in 2018–2020 (*p = *0.040), in patients older than 80 years from 19.2 to 12.3%, respectively (*p = *0.042), and for ASA III–IV patients from 24.3 to 13.7% (*p = *0.003), but not in males (*p = *0.341). The cumulative 1-year mortality risk declined significantly from 16.2% in 2010–12 to 10.4% in 2018–20 in females (*p = *0.009), and ASA III–IV patients from 31.2 to 20.3%, respectively (*p = *0.006), but there were no significant differences between the two periods in males (*p = *0.545) or patients older than 80 years (*p = *0.053). There were no significant differences between fracture types at any postoperative time analyzed (all *p > *0.05).

**Table 2:** Cumulative brute mortality in each cohort

Mortality	2010–12 *n = *591	2018–20 *n = *642	*p* value
In-hospital, overall	14/591 (2.5%)	13/642 (2.0%)	0.339
Female	8/445 (1.8%)	7/450 (1.5%)	0.491
Male	6/146 (4.1%)	6/192 (3.1%)	0.421
Age 65–79	4/420 (0.9%)	4/424 (0.9%)	0.632
Age ≥ 80	10/171 (5.8%)	9/218 (4.1%)	0.291
ASA I–II	2/402 (0.5%)	0/401 (0%)	0.497
ASA III–IV	12/189 (6.3%)	13/241 (5.4%)	0.413
Charlson ≤ 2	2/254 (0.8%)	1/244 (0.4%)	0.515
Charlson > 2	12/578 (2.1%)	12/398 (3.0%)	0.234
At 30 days, overall	49/591 (8.3%)	35/642 (5.5%)	0.031*
Female	39/445 (8.8%)	25/450 (5.5%)	0.040*
Male	10/146 (6.8%)	10/192 (5.2%)	0.341
Age 65–79	18/420 (4.3%)	8/424 (1.9%)	0.033*
Age ≥ 80	33/171 (19.2%)	27/218 (12.3%)	0.042*
ASA I–II	3/402 (0.7%)	2/401 (0.5%)	0.501
ASA III–IV	46/189 (24.3%)	33/241 (13.7%)	0.003*
Charlson ≤ 2	2/254 (0.8%)	1/244 (0.4%)	0.673
Charlson > 2	47/578 (8.1%)	34/398 (8.5%)	0.453
At 1 year, overall	95/591 (16.1%)	77/642 (11.9%)	0.023*
Female	71/445 (16.2%)	47/450 (10.4%)	0.009*
Male	23/146 (15.7%)	30/192 (15.6%)	0.545
Age 65–79	60/420 (14.3%)	47/450 (10.4%)	0.052
Age ≥ 80	35/171 (20.4%)	30/218 (13.7%)	0.053
ASA I–II	36/402 (8.9%)	28/401 (6.9%)	0.183
ASA III–IV	59/189 (31.2%)	49/241 (20.3%)	0.006*
Charlson ≤ 2	33/254 (12.9%)	32/244 (13.1%)	0.536
Charlson > 2	62/578 (10.7%)	45/398 (11.3%)	0.426

Data as *n* (%)

*Significant *p* value

In multivariate analysis (Table 3), the only significant predictor of in-hospital mortality was ASA III–IV in both studied periods (*p = *0.021 in 2010–12, and 0.048 in 2018–20), but it is to be noted that the Charlson index > 2 was not a predictor in either period (*p = *0.321 and 0.425, respectively). For 30-day mortality, multivariate analysis showed in both 2010–12 and 2018–20 periods that male gender (*p = *0.028 and 0.016, respectively), age older than 80 years (*p = *0.008 and 0.037, respectively), and ASA category III–IV (*p = *0.025 and 0.017, respectively) were significant predictors, although the Charlson index was not significant (*p = *0.076 and 0.059, respectively). In multivariate analysis for 1-year mortality, male gender (*p = *0.022 in 2010–12, and 0.038 in 2018–20), age older than 80 years *p = *0.009 and 0.031, respectively), and Charlson index > 2 (*p = *0.041 and 0.040, respectively) were significant predictors, but not ASA category III–IV (*p = *0.082 and 0.377, respectively). Despite the significant declines in 30-day and 1-year mortalities from 2010–12 to 2018–20, there was no improvement compared with the mortality in the general population of similar gender and age (Table 4). Based on the standardized reference population, the observed 1-year mortality risks during 2010–12 (SMR 1.51, 95% CI 1.17–1.89) and 2018–20 (SMR 1.33, 95% CI 1.25–1.61) were higher than expected in the same periods.

**Table 3:** Multivariate analysis for mortality according to cohorts (2010–12 vs 2018–20)

Mortality	In-hospital		30 days		1 year	
	HR (95% CI)	*p*	HR (95% CI)	*p*	HR (95% CI)	*p*
Males						
2010–12	1.5 (0.9–2.1)	0.394	1.7 (1.1–2.2)	0.028*	2.4 (2.2–2.9)	0.022*
2018–20	1.4 (1.2–2.0)	0.421	1.5 (1.0–2.1)	0.016*	2.3 (2.0–2.7)	0.038*
*p*	0.264		0.074		0.061	
Age > 80						
2010–12	1.3 (1.0–3.9)	0.121	2.4 (2.0–3.3)	0.008*	2.1 (1.3–2.9)	0.009*
2018–20	1.6 (0.9–1.8)	0.310	2.6 (1.9–3.0)	0.037*	1.8 (1.5–2.3)	0.031*
*p*	0.168		0.061		0.041*	
ASA III–IV						
2010–12	1.6 (1.2–1.9)	0.021*	2.1 (1.3–2.4)	0.025*	1.8 (1.0–2.1)	0.082
2018–20	1.4 (1.1–1.9)	0.048*	1.7 (1.1–1.9)	0.017*	1.6 (0.9–2.0)	0.377
*p*	0.168		0.042*		0.532	
Charlson > 2						
2010–12	1.5 (0.8–2.0)	0.321	1.7 (0.9–2.3)	0.076	1.3 (1.1–1.8)	0.041*
2018–20	1.3 (0.9–1.9)	0.425	1.4 (0.8–1.9)	0.059	1.2 (1.0–1.9)	0.040*
*p*	0.241		0.632		0.589	
BMI						
2010–12	1.1 (0.3–1.7)	0.361	1.1 (0.7–1.8)	0.394	1.7 (0.8–2.4)	0.513
2018–20	1.0 (0.6–1.6)	0.187	1.0 (0.6–1.6)	0.542	1.9 (0.9–2.1)	0.681
*p*	0.847		0.824		0.365	
Cervical fracture						
2010–12	1.4 (0.6–2.0)	0.432	1.3 (0.8–1.9)	0.595	1.3 (1.0–6.9)	0.728
2018–20	1.3 (0.8–2.1)	0.632	1.1 (0.8–1.8)	0.438	1.0 (0.5–1.7)	0.426
*p*	0.618		0.714		0.058	
TTS > 48 h						
2010–12	1.3 (0.9–3.4)	0.520	1.2 (0.8–1.6)	0.549	1.5 (0.2–1.8)	0.594
2018–20	1.2 (0.9–2.5)	0.106	1.0 (09–1.7)	0.463	1.3 (0.9–1.8)	0.317
*p*	0.624		0.657		0.432	

*HR* hazard ratio, *CI* confidence interval, *TTS* time to surgery

*Significant *p* value

**Table 4:** Standardized mortality ratio (SMR) at 1 year

Subgroups	2010–12	2018–20
	SMR (95% CI)	SMR (95% CI)
Overall	1.51 (1.37–1.69)	1.33 (1.25–1.56)
Females	1.65 (1.34–1.86)	1.22 (1.17–1.58)
Age 65–79	1.28 (1.04–1.60)	1.05 (1.02–1.59)
Age ≥ 80	2.16 (1.84–2.72)	1.26 (1.21–1.75)
Males	1.59 (1.42–1.73)	1.29 (1.11–1.39)
Age 65–79	1.17 (1.09–1.93	1.10 (1.09–1.49)
Age ≥ 80	1.84 (1.72–2.04)	1.31 (1.20–1.58)

*CI* confidence interval

## Discussion

The initiatives introduced during the last decade to improve the care of elderly patients with hip fractures, especially co-management with geriatricians and shorter time to surgery, have had a positive impact on mortality within 1-year postoperatively. However, the postoperative survival in these patients is still lower than in the general population of similar age and gender.

A significant decrease over time of the in-hospital mortality has been reported in the literature, from 12% in the 1990s [18] to 3.5%–4.7% in the 2000s [19]. The in-hospital mortality in the present study agrees with the risk about 2% reported currently [20], and female gender was not a significant predictor, while female was significant in the cohort by Forni et al. [20]. A recent meta-analysis [21] indicated reduced mortality for patients operated within 24 h compared with those operated within 36 h. Contrary, other recent study [22]) reported that early surgery within 48 and 72 h was significantly associated with a lower in-hospital mortality in patients older than 85 years. In a prior study [23], we found that patients with severe active comorbidities could benefit from surgery delayed more than 2 days. On the other hand, the co-management between surgeons and geriatricians has showed a significant reduction of the in-hospital mortality [24].

The in-hospital mortality risk remained unchanged over the last decade, and this was influenced by older mean age and more patients with comorbidities and high surgical risk as measured by the ASA in the last 3 years studied. However, significant improvements in mortality at 30-day and 1-year postoperatively were observed in the present study. Age and male gender influenced mortality, although the proportion of patients older than 80 years were gradually highest. Moreover, together with an increase over time in age there was also an increase in patient comorbidity. It is to be noted that the comorbidity as measured by the ASA was a mortality predictor at in-hospital and 30 days, but not at 1 year. On the contrary, comorbidity measured by the Charlson index was a predictor of 1-year mortality. This could be because the ASA assesses comorbidity for an immediate surgical risk, while the Charlson index assesses health status with respect to concomitant chronic diseases that can worsen over time. The improvement in 30-day mortality over the last decade was likely the result of better clinical perioperative management and faster regain of weight-bearing, and the improvement in 1-year mortality was the result of an improvement in the care for these patients in the public health system.

The evolution over time of 30-day mortality is controversial in the literature. A Danish trend study found no significative change in 30-day mortality between 1980 and 1990 [7], with a risk of 9%, while other Danish study based on national registry reported similar risk of 9% between 2000 and 2014 [5]. Contrary, a recent study on the Swedish national registry [12] found the 30-day mortality increased between 1998 and 2017 from 4.3% to 6.2% in women and from 8.4 to 11.1% in men, which was attributed to the fact that the length of stay had decreased. The most recent studies found a decline from 12 to 7% between 2000 and 2016 [6], and risks between 2018 and 2020 ranged from 5 to 7% [20]. An English report found a 30-day mortality risk of 1.2% during 2009–10 [25]. Like us, some of these more recent studies reported a higher 30-day mortality risk in males compared with females between 2010 and 2017 [11, 24, 26], while others found that male was not significant predictor in 2014 [20]. In the present study, the 30-day mortality decreased significantly from 2010 to 2020, despite an increase over time in the mean age of patients and their comorbidities.

In most studies of patients treated in past and recent decades, 1-year mortality remained unchanged despite multi-disciplinary cares [27]. In the present study, 1-year mortality significantly declined over the last decade, and the risk at 1 year was substantially lower than those reported in past decades [6, 28]. The 1-year mortality is high over time in the literature, with risks about 29% in the 1960s [3], 23% in the 1980s [29], and 26% in the 1990s [7, 28]. In the most recent studies, no significant changes in the last 20 years have been reported with risks maintained about 22% [2, 6, 11]. Like us, the most common predictors reported were male, age > 80 years, and Charlson index > 2 [30]. Nevertheless, mortality risks are influenced by various factors. Kjaerevik et al. [11] included 18 factors in a study of mortality after hip fracture. Sheehan et al. [31] identified 39 patient-factors that could be associated with mortality after hip fracture. Compared with the general population of similar age and gender, hip fracture patients had 1-year mortality risk 16 times higher in females, and 12 times higher in males during the 1990s [32], with a SMR of 2.4 increased risk in females and 3.5 in males in 2010 [33], and of 3.5 for both genders in 2018 [11]. However, this decrease did not translate into increased survival as compared with that in the general population of similar gender and age. SMR showed a higher mortality risk over the last decade, of 1.91 in 2010 and 1.84 in 2020, which was not a substantial difference. The higher relative excess mortality at 1-year after hip fracture, as compared with the general population, probably reflects the frailty and multi-morbidity in the elderly patients who sustain a hip fracture.

To our knowledge, this is the study to analyze mortality change over time after hip fracture in elderly patients using more recent patient cohorts. Although this was a retrospective study, perioperative data had been collected prospectively without the problem of variable completeness of reporting of population-based or administrative register studies. However, the study has several limitations. The sample of patients was relatively small as it was based on a single hospital. However, the sample was representative of patients treated in routine clinical practice, there were a complete follow-up of patients without loss of their data, and reliable identification of deaths. On the other hand, the results of this single institution study may not be extrapolated to other populations, since mortality may be associated with multiple factors including lifestyle, socio-economic status and facilities to live at a nursing home. A recent study [34] found that 30-day mortality varied from 5 to 9% among 32 public hospitals in the same country. The authors suggested that this could be due to a varied adherence to current clinical guidelines.

In conclusion, the important evolution achieved in the last decade for the management of patients with hip fracture surgery has led to a significant decline in 1-year mortality, but the 1-year mortality remains significantly higher compared to the general population of similar age and gender.

## Data Availability

Data are available by requesting the corresponding author.
